# A Case of Recurrent Pemphigus Foliaceus Following Noncompliance to Medication

**DOI:** 10.1002/ccr3.9622

**Published:** 2024-11-22

**Authors:** Masab Ali, Muhammad Husnain Ahmad, Ali Imran, Uswa Ahmad, Rehan Naseer Ahmad

**Affiliations:** ^1^ Department of Dermatology Punjab Medical College Faisalabad Pakistan; ^2^ Department of Medicine Tentishev Satkynbai Memorial Asian Medical Institute Kant Kyrgyzstan; ^3^ Department of Medicine King Edward Medical University Lahore Pakistan; ^4^ Department of Medicine Aziz Fatimah Medical and Dental College Faisalabad Pakistan

**Keywords:** autoimmune diseases, azathioprine, blisters, dexamethasone‐cyclophosphamide pulse, immunosuppressive therapy, pemphigus

## Abstract

Pemphigus foliaceus (PF) is a rare autoimmune blistering disorder requiring consistent immunosuppressive therapy for management. A 66‐year‐old male with a history of PF presented with worsening blisters and erosions after discontinuing medication. The patient had flaccid bullae and erosions on the face, scalp, chest, and back. Histopathology confirmed PF. Treatment with oral prednisolone, azathioprine, and reinitiation of dexamethasone‐cyclophosphamide pulse (DCP) therapy led to disease remission. This case underscores the importance of adherence to immunosuppressive therapy in PF management. It also highlights the role of affordable treatment regimens in ensuring patient compliance and successful outcomes.

**Summary**

- Adherence to a well‐structured immunosuppressive regimen is essential for managing pemphigus foliaceus.
- Noncompliance, often because of affordability, can worsen symptoms.
- The phased approach of dexamethasone‐cyclophosphamide pulse therapy, combined with prednisolone and azathioprine, improves patient outcomes.
- Consistent follow‐up and patient support are key to improving adherence and preventing relapse.

## 1 Introduction

Pemphigus foliaceus (PF) is a rare autoimmune blistering disorder characterized by superficial blistering of the skin without mucosal involvement [1]. It is usually diagnosed by investigations such as histopathology after a skin biopsy, perilesional skin immunofluorescence, and enzyme‐linked immunosorbent assay (ELISA) of anti‐desmoglein 1 (Dsg1) antibody (Ab) [2]. Subcorneal acantholysis is a characteristic feature which differentiates it from pemphigus vulgaris (PV), the common pemphigus subtype, which involves the suprabasal level [2, 3]. Effective management typically involves immunosuppressive therapy such as steroids, azathioprine, and cyclophosphamide [4]. This case report highlights the consequences of discontinuing dexamethasone‐cyclophosphamide pulse (DCP) therapy and the successful reintroduction of treatment, resulting in disease remission.

## 2 Case Presentation

A 66‐year‐old male presented with worsening skin blisters and erosions over the past 2 months. The patient had a history of PF, diagnosed 1 year ago. Initially, the patient presented with multiple blisters and erosions on the skin and was treated with monthly DCP therapy and oral plus topical steroids. However, he discontinued the DCP therapy and ceased taking medications because of affordability issues. Table 1 summarizes the timeline of events that happened to this patient.

**TABLE 1:** Timeline of Events.

Date	Event
1 year ago	Initial presentation with multiple blisters and erosions.
8 months ago	Diagnosed with PF, started on monthly DCP therapy and oral steroids.
6 months ago	Discontinued pulse therapy and ceased taking medications.
2 months ago	Worsening of blisters and erosions in seborrheic areas.
Current	Presented with worsening of symptoms, the patient was reinitiated on DCP therapy along with steroids and steroid‐sparing drugs.

**FIGURE 1:**
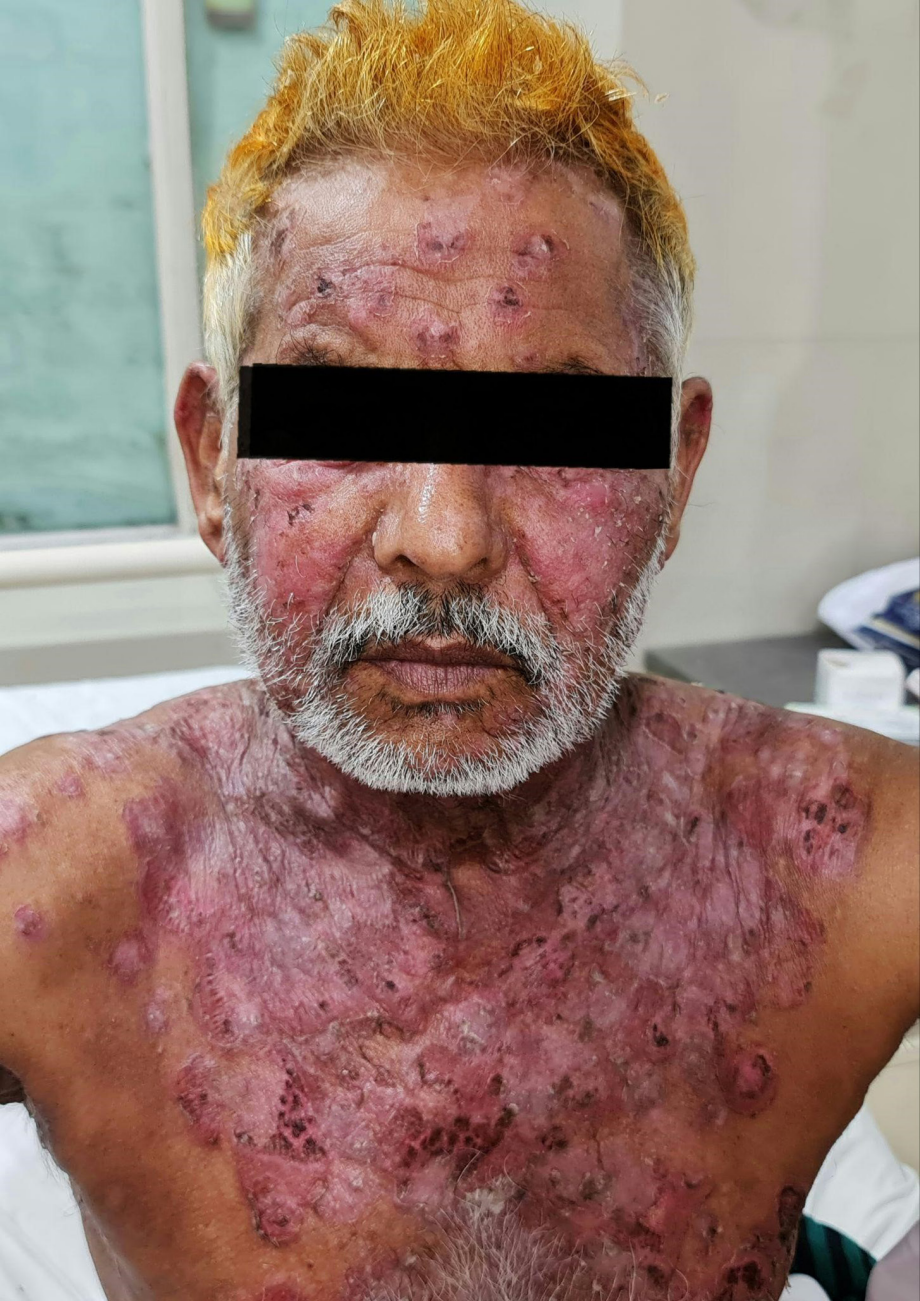
Erosions on chest, neck, and face of the patient.

**FIGURE 2:**
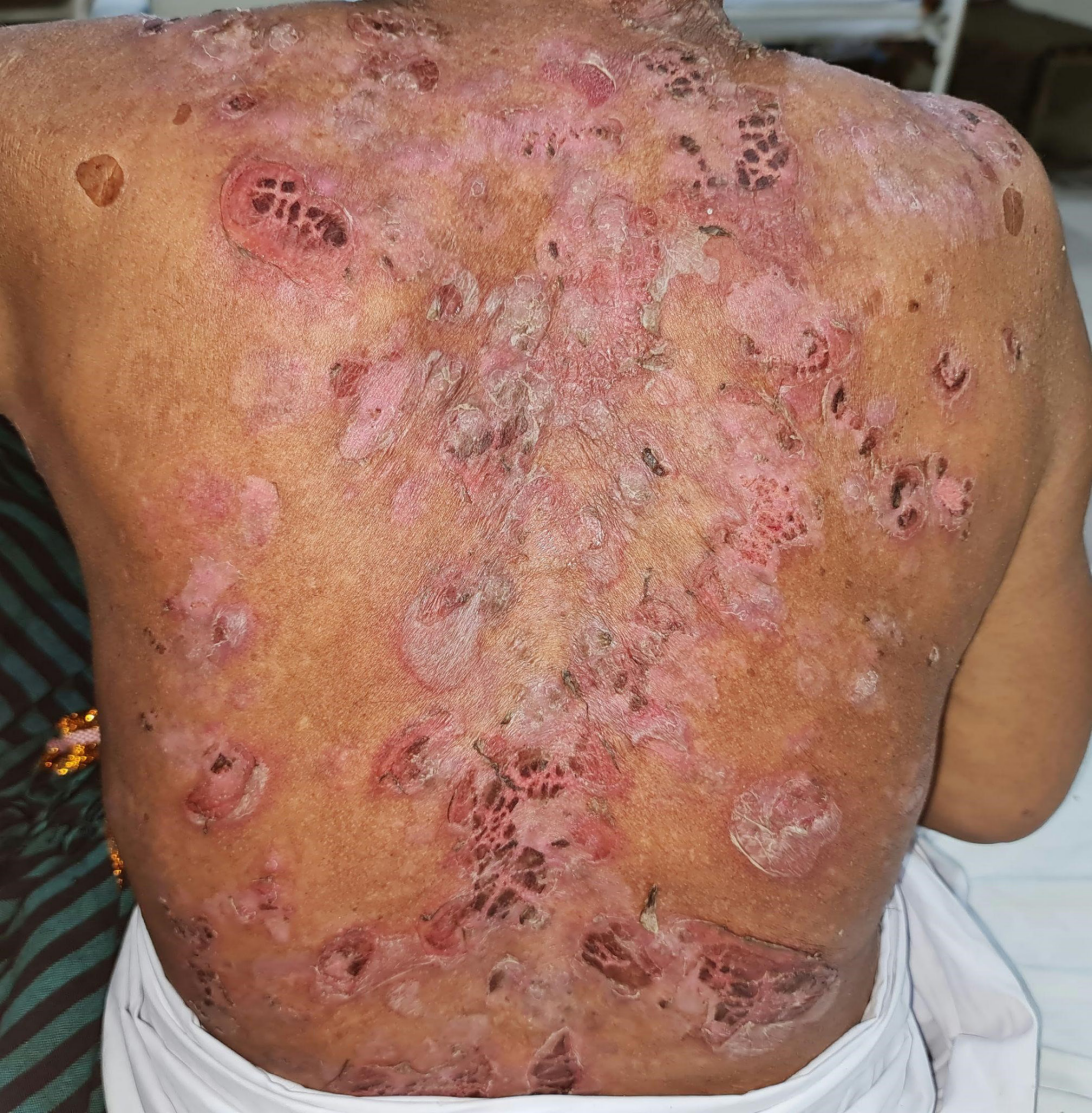
Ruptured flaccid bullae leaving behind erosions on the back of the patient.

On physical examination (see Figures 1 and 2), the patient exhibited multiple flaccid bullae and erosions on the face, scalp, chest, and back, with no mucosal involvement. The pattern of the disease is very significant and most likely indicates PF [2].

## 3 Differential Diagnosis

Differential diagnosis of PV and PF was made according to the history and examination of the patient.

## 4 Investigations

At the time of presentation, his vital signs were as follows: blood pressure 120/70 mmHg, heart rate 110 bpm, temperature 100°F, respiratory rate 12/min, and oxygen saturation 97% at room air. Baseline laboratory tests were performed. The complete blood count (CBC) revealed hemoglobin of 11.3 g/dL, total leukocyte count of 10,300/μL, and platelet count of 248,000/μL. Liver function tests (LFTs) showed bilirubin at 0.4 mg/dL, alanine transaminase at 34 U/L, and alkaline phosphatase at 240 U/L. Renal function tests (RFTs) indicated urea at 38 mg/dL and creatinine at 1.0 mg/dL. Urinalysis (UA) was normal, with no protein, glucose, or red blood cells detected. Serum electrolytes showed sodium at 137 mmol/L and potassium at 4.9 mmol/L.

**FIGURE 3:**
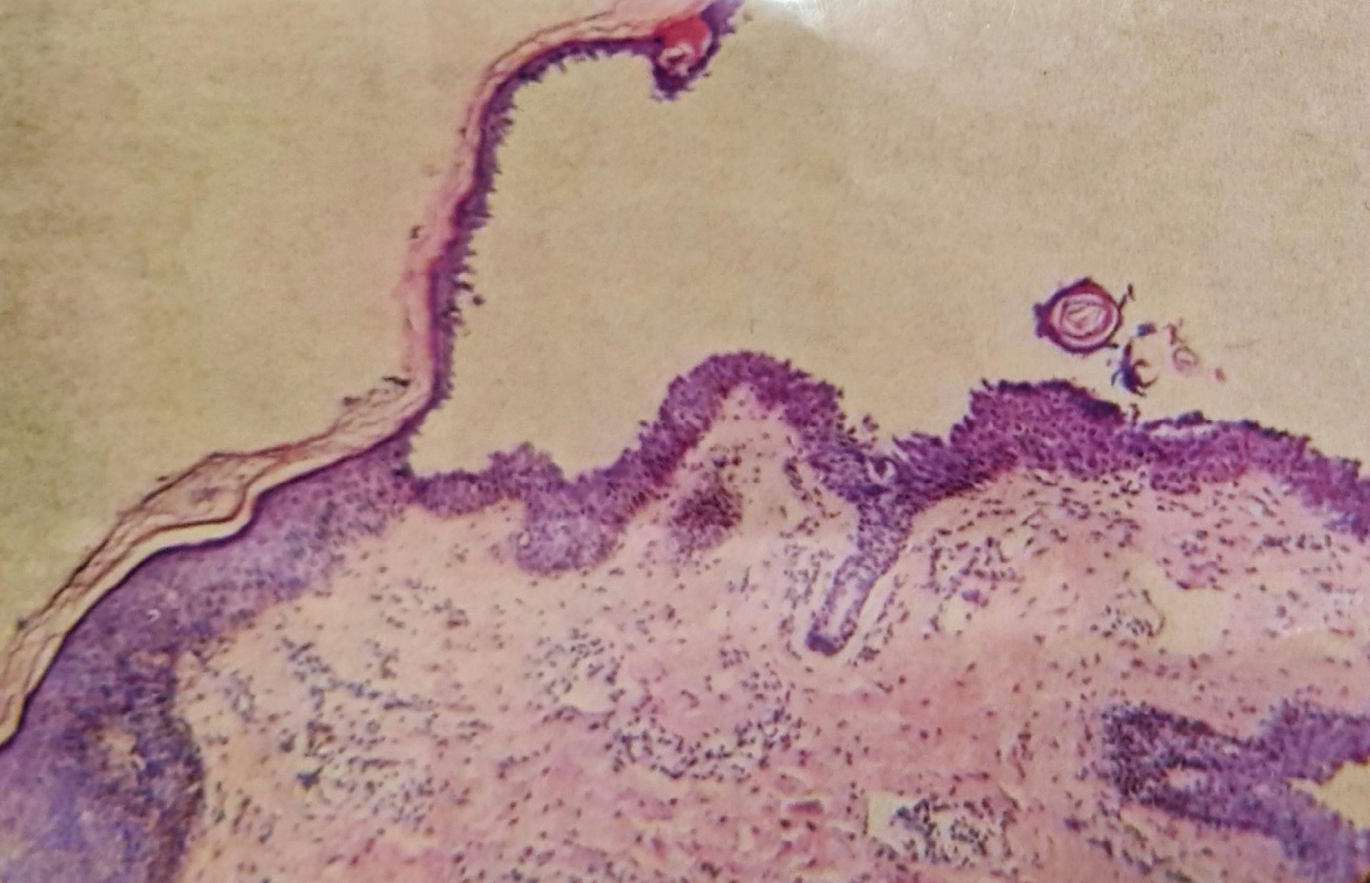
The histopathology slide shows an intraepidermal split at the level of the subcorneum and granular layer. The deeper dermis is intact.

**FIGURE 4:**
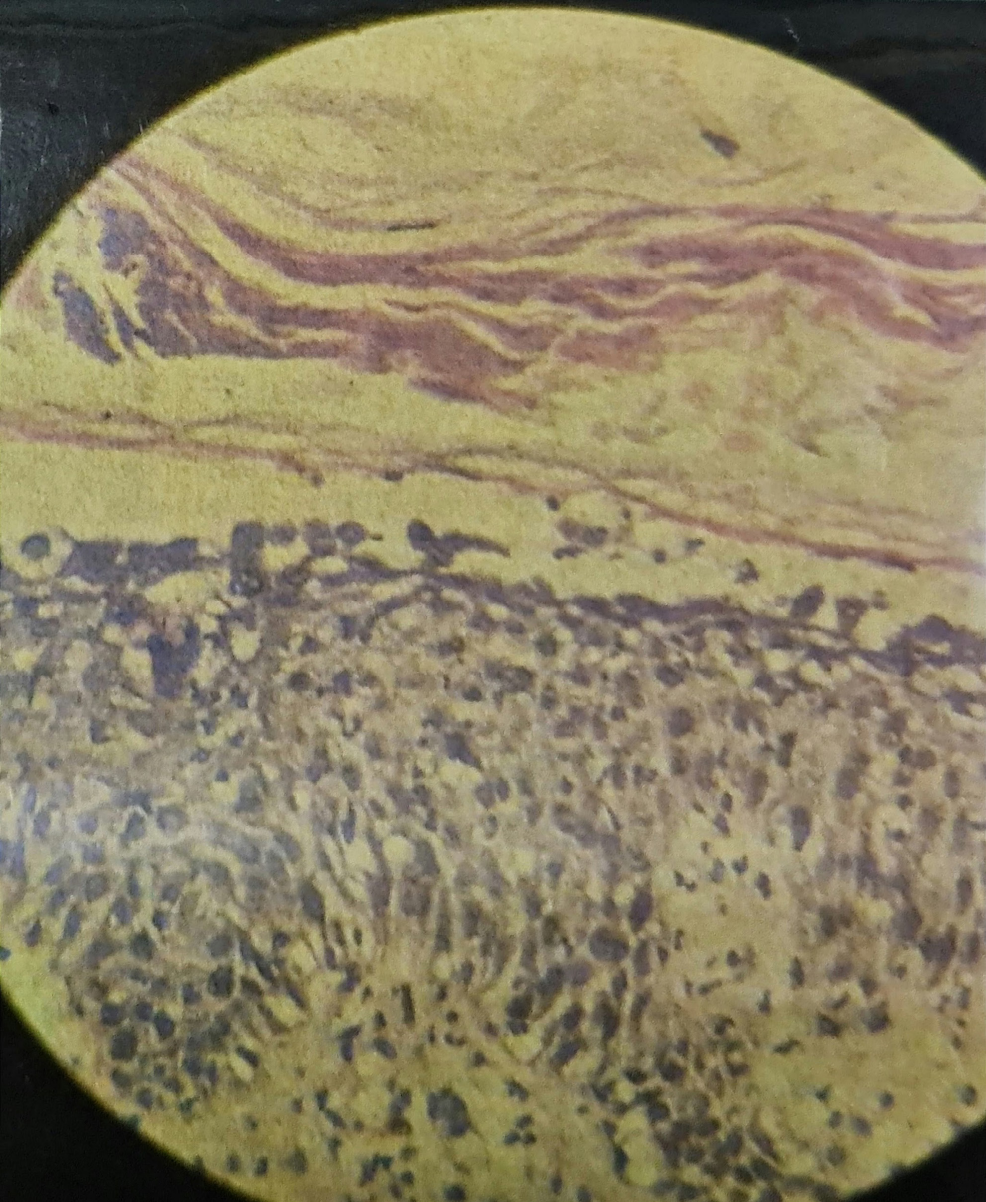
The slide shows spongiosis with focal basal layer vacuolation, dermal edema with numerous neutrophils, and lymphocytes in the papillary dermis.

A biopsy was taken from the back of the neck to rule out differentials. Histopathology showed a normal epidermis with focal hyperkeratosis, an intraepidermal split at the level of the subcorneum and granular layer (see Figure 3), and a blister cavity containing numerous acantholytic cells. The epidermis beneath the blisters showed spongiosis with focal basal layer vacuolation and dermal edema (see Figure 4) with numerous neutrophils and lymphocytes in the papillary dermis. These histopathological features, along with clinical symptoms, confirmed the diagnosis of PF.

## 5 Management

The patient had previously discontinued medication because of cost concerns. Upon reinitiation, the health‐care team enrolled the patient in a medication assistance program, resolving the cost issue. The patient was started on oral prednisolone 60 mg/day and oral azathioprine 50 mg/day, along with calcium and potassium chloride supplementation to address steroid‐induced electrolyte imbalances. Treatment was organized into a phased schedule to achieve remission and maintain long‐term disease control. Initially, the patient received dexamethasone (100 mg in 500 mL 5% dextrose) over 2 h for three consecutive days, along with 500 mg of cyclophosphamide on day 1 of each pulse. This DCP is repeated every 4 weeks [5]. Throughout the treatment, the dose of prednisolone was gradually tapered at each visit based on the patient's response and improvement in symptoms. This approach helped reduce the risks associated with long‐term steroid use while maintaining disease control. The patient was also advised to apply topical salicylic acid with liquid paraffin to the lesions and to bathe daily.

## 6 Outcome and Follow‐Up

Following this treatment, the patient's disease entered remission, characterized by the absence of new lesions and the healing of existing ones. Regular follow‐up visits are scheduled every month for continued‐DCP therapy and laboratory monitoring, including CBC, LFT, UA, and RFT, to assess potential side effects from long‐term cyclophosphamide and steroid use. Adjustments to the treatment plan will be made as necessary.

This regimen will continue for 6 months, after which, if remission is sustained, the monthly pulses will be discontinued [5]. However, the oral steroid‐sparing therapy will continue for another 9 months [5]. Regular follow‐up is crucial to maintaining long‐term disease control, ensuring patient compliance, and preventing relapses in autoimmune conditions such as PF.

## 7 Discussion

PF presents with superficial flaccid bullae that rupture easily, leading to erosions, particularly in seborrheic areas [2]. Diagnosis can be made through a skin biopsy, which reveals characteristic histopathological features [3, 6]. Immunofluorescence is not always necessary, as histopathology can differentiate PF from PV by showing an intraepidermal split at the level of the subcorneum and granular layer [3]. PF predominantly affects the stratum corneum, unlike PV, which affects deeper skin layers near the dermis and involves the oral cavity [7]. PV produces anti‐Dsg1 Ab and Dsg3 Ab, with Dsg3 present in the oral cavity, leading to oral involvement. In contrast, PF usually produces Ab only against Dsg1, which is not present in oral mucosa, explaining the lack of mucosal involvement in PF.

This case underscores the importance of adherence to immunosuppressive therapy in managing the condition. The patient's discontinuation of DCP therapy led to a significant worsening of symptoms, which were subsequently controlled upon reinitiation of the therapy. The strengths of this case include the detailed histopathological analysis and clear demonstration of the clinical improvement with resumed treatment. Literature suggests that consistent immunosuppressive therapy is crucial in maintaining remission in PF. Cyclophosphamide is a potent immunosuppressive agent that has shown efficacy in inducing and maintaining remission in PF [8]. It works by reducing the immune system's attack on the skin, thereby preventing the formation of new blisters and promoting the healing of existing lesions. The combination of cyclophosphamide with steroids, such as prednisolone, enhances the therapeutic effect and helps in faster control of the disease [9]. Azathioprine, added to the treatment regimen, is an effective and more affordable steroid‐sparing agent. It works by inhibiting DNA synthesis in rapidly dividing cells, including those of the immune system, thus reducing the autoimmune response [10, 11]. Rituximab, a potent anti‐B‐cell agent, has demonstrated efficacy in PF by targeting B cells and reducing autoantibody production [2]. The patient's financial limitations precluded the use of rituximab, which has shown superior outcomes in some studies but is costly [12].

Monitoring disease remission typically involves checking for new lesion formation. A limitation of this case is the lack of long‐term follow‐up data after therapy was reinitiated. Dsg1 Ab ELISA can be used to monitor disease remission in addition to clinical evaluation [13, 14]. However, in this patient, only clinical assessment was performed because of the visible healing of lesions and the absence of new lesions, indicating remission. It represents a limitation of the report, as Ab titers were not measured.

## 8 Conclusion

This case highlights the importance of immunosuppressive therapy in managing PF. Discontinuation can lead to severe symptom exacerbation, whereas reinitiation effectively induces remission. Patient compliance and access to affordable treatment are crucial for success. A phased regimen of cyclophosphamide and prednisolone, with maintenance DCP therapy and azathioprine as a steroid‐sparing agent, proved effective. The structured, phased approach ensured long‐term disease control and improved patient outcomes.

## Author Contributions

**Masab Ali:** conceptualization, data curation, formal analysis, investigation, project administration, resources, supervision, validation, visualization, writing – original draft, writing – review and editing. **Muhammad Husnain Ahmad:** visualization . **Ali Imran:** validation, visualization, writing – review and editing. **Uswa Ahmad:** resources, visualization, writing – review and editing. **Rehan Naseer Ahmad:** validation, visualization.

## Consent

Written informed consent was obtained from the patient to publish this report in accordance with the journal's patient consent policy.

## Conflicts of Interest

The authors declare no conflicts of interest.

## Data Availability

The data is available upon reasonable request from the corresponding author.

## References

[1] M. Hertl and C. Veldman , “Pemphigus—Paradigm of Autoantibody‐Mediated Autoimmunity,” Skin Pharmacology and Applied Skin Physiology 14, no. 6 (2001): 408–418. 11598441 10.1159/000056375

[2] T. Burns , S. M. Breathnach , N. Cox , and G. Christopher , Rook's Textbook of Dermatology (John Wiley & Sons, 2024), 10.1002/9781119709268.

[3] N. Stumpf , S. Huang , L. D. Hall , and S. Hsu , “Differentiating Pemphigus Foliaceus From Pemphigus Vulgaris in Clinical Practice,” Cureus 11 (2021): e17889, 10.7759/cureus.17889. PMC8437008 34548989

[4] H. C. Nousari , A. Sragovich , A. Kimyai‐Asadi , and G. J. Anhalt , “Clinical Improvement in Patients With Pemphigus Foliaceus Following Cyclophosphamide Therapy,” Journal of the American Academy of Dermatology 40, no. 5 Pt 1 (1999): 734–736.

[5] S. Mustafi , R. Sinha , S. Hore , S. Sen , S. Maity , and P. Ghosh , “Pulse Therapy: Opening New Vistas in Treatment of Pemphigus,” Journal of Family Medicine and Primary Care 8, no. 3 (2019): 793–798, 10.4103/jfmpc.jfmpc_114_19. PMC6482734 31041203

[6] D. Flood , A. Lezanski‐Gujda , and N. R. Miletta , “Diagnosing Pemphigus Foliaceus: A Rare Blistering Disease Masquerading as a Common Dermatologic Disorder,” Military Medicine 184, no. 5–6 (2019): e455. 30215775 10.1093/milmed/usy224

[7] E. Sokol , D. Kramer , G. F. H. Diercks , et al., “Large‐Scale Electron Microscopy Maps of Patient Skin and Mucosa Provide Insight Into Pathogenesis of Blistering Diseases,” Journal of Investigative Dermatology 135, no. 7 (2015): 1763–1770, 10.1038/jid.2015.109. 25789704

[8] M. A. Cohen , J. J. Cohen , and F. A. Kerdel , “Immunoablative High‐Dose Cyclophosphamide Without Stem Cell Rescue in Pemphigus Foliaceus,” International Journal of Dermatology 41, no. 6 (2002): 340–344. 12100688 10.1046/j.1365-4362.2002.01407.x

[9] M. Salińska , K. Trambowicz , J. Narbutt , A. Woźniacka , and A. Lesiak , “Combination Therapy With Prednisone and Cyclophosphamide in a 14‐Year‐Old Boy With Pemphigus Foliaceus—A Case Report,” Dermatology Review 104, no. 3 (2017): 324–330, 10.5114/dr.2017.68779.

[10] S. Beissert , T. Werfel , U. Frieling , M. Bohm , M. Sticherling , and W. Sterry , “A Comparison of Oral Prednisolone and Prednisolone Plus Azathioprine in the Treatment of Pemphigus,” Archives of Dermatology 132, no. 6 (1996): 646–652.

[11] S. Baum , N. Sakka , O. Artsi , A. Barzilai , and H. Trau , “The Role of Adjuvant Therapy in Pemphigus: A Systematic Review and Meta‐Analysis,” Journal of the American Academy of Dermatology 69, no. 4 (2013): 603–610. 26088689 10.1016/j.jaad.2015.04.038

[12] S. Das , K. Agarwal , S. Singh , D. Halder , S. Sinha , and A. De , “A Comparative Study to Evaluate the Efficacy and Cost of Rituximab Versus Dexamethasone Cyclophosphamide Pulse in Patients of Pemphigus Vulgaris,” Indian Journal of Dermatology 66, no. 2 (2021): 223, 10.4103/ijd.IJD_306_20. PMC8208287 34188295

[13] C. Abasq , H. Mouquet , D. Gilbert , et al., “ELISA Testing of Anti‐Desmoglein 1 and 3 Antibodies in the Management of Pemphigus,” Archives of Dermatology 145, no. 5 (2009): 529–535, 10.1001/archdermatol.2009.9. 19451496

[14] D. F. Murrell , S. Peña , P. Joly , et al., “Diagnosis and Management of Pemphigus: Recommendations of an International Panel of Experts,” Journal of the American Academy of Dermatology 82, no. 3 (2020): 575, 10.1016/j.jaad.2018.02.021. 29438767 PMC7313440

